# Development and evaluation of an internet- and mobile-based intervention for individualized return to work planning after inpatient rehabilitation - Study protocol for a randomized-controlled-trial

**DOI:** 10.1016/j.invent.2024.100721

**Published:** 2024-02-01

**Authors:** Adina Kreis, Anna Gomes, Angeliki Tsiouris, Manfred E. Beutel, Christian Ruckes, Ingo Dahn, Annika Schiller, Guido Loy, Hiltrud Zajac, Gregor Kosmuetzky, Patrick Ziser, Eckard Sträßner, Vera Schneider, Thomas Wilde, Martin Leber, Hannah Schäfer, Rebecca Kilian, Rüdiger Zwerenz

**Affiliations:** a Department of Psychosomatic Medicine and Psychotherapy, University Medical Center Mainz, Johannes Gutenberg-University, Untere Zahlbacher Str. 8, 55131 Mainz, Germany; b Interdisciplinary Center for Clinical Trials, University Medical Center Mainz, Langenbeckstraße 1, 55131 Mainz, Germany; c Virtual Campus Rhineland-Palatinate, Erwin-Schrödinger-Straße, 67663 Kaiserslautern, Germany; d Psychosomatic Clinic, Campus Bad Neustadt, Kurhausstraße 31, 97616 Bad Neustadt, Germany; e Rehabilitation Center Bad Driburg - Clinic Berlin, Brunnenstraße 11, 33014 Bad Driburg, Germany; f Rehabilitation Center Schömberg - Clinic Black Forest, Römerweg 50, 75328 Schömberg, Germany; g St. Franziska Stift - Rehabilitation Clinic Bad Kreuznach, Franziska-Puricelli-Straße 3, 55543 Bad Kreuznach, Germany

**Keywords:** Internet- and mobile-based intervention, Occupational e-mental health, Rehabilitation aftercare, Return to work, Randomized-controlled-trial, Study protocol

## Abstract

**Background:**

Following discharge, it is crucial for patients to transfer intentions and action plans from inpatient rehabilitation into everyday life. This ensures their reintegration into social and working life and prevents economic costs due to sick leave or reduced earning capacity pension. However, most established aftercare programs do not specifically address occupational problems or challenges during occupational measures such as graded return to work. The aim of this study is to evaluate the efficacy of the low-threshold online self-help intervention *marena* (Meine Arbeitsbezogene Reha-Nachsorge - My Work-related Rehabilitation Aftercare) to support return to work.

**Methods:**

A two-arm randomized-controlled-trial (RCT) will be conducted. A total of *N* = 400 rehabilitation inpatients across different indication areas (psychosomatic, orthopedic, or cardiologic) aged 18 to 65 years with a planned return to work after medical rehabilitation, have a heightened social-medical risk and private internet access and are insured with the German Pension insurance or statutory health insurance, will be recruited in four medical and psychosomatic clinics in Germany. Participants will be allocated to either the intervention (IG) or the control group (CG). In a stepped-care model, participants of the IG will receive access to the non-guided internet- and mobile-based intervention *marena* (IG subgroup 1) or *marena* in combination with *GSA-Online plus* (IG subgroup 2), a guided psychodynamic internet-based intervention that has proven effective in two trials regarding occupational and health objectives. Based on a priori defined indication criteria, clinic staff will recommend either IG subgroup 1 or IG subgroup 2. The CG will receive optimized treatment as usual with access to a survey feature within *marena*. The primary outcome will be work status after 6 months (T2) and 12 months (T4). The endpoint at 12 months (T4) after discharge from inpatient rehabilitation will be considered as secondary endpoint. Work status is defined as positive if the participant is working and has ≤ 6 weeks of sick leave at T2 and ≤ 12 weeks of sick leave at T4. Secondary outcomes include successful completion of graded return to work, successful application for benefits for participation in working life, current work ability, social-medical risk, subjective prognosis of future employment, quality of life, somatic symptoms, coping, social support, depression, anxiety, and psychosocial stress.

**Discussion:**

This study will contribute to the evidence concerning efficacy of online aftercare interventions. If proven efficacious, *marena* could provide an individualized and adaptable self-help approach to promote return to work following inpatient rehabilitation.

## Highlights

• Internet-based aftercare interventions promise to complement inpatient rehabilitation.
• The self-guided intervention *marena* provides personalized aftercare plans.
• In a stepped-care approach a guided writing intervention provides additional support.
• The effectiveness for return to work is evaluated in a randomized controlled trial.

## 1 Background

Medical rehabilitation, as defined by the German Pension Insurance, aims to maintain work ability partially or completely and to (re-)integrate patients with chronic illness or disability into social and working life (Deutsche Rentenversicherung Bund, 2022). In Germany, inpatient rehabilitation is considered when outpatient rehabilitation is not possible or sufficient. In the clinic, patients receive support in managing their illnesses or disabilities, are formally incapacitated to work, and are distanced from daily burdens (Linden, 2014). Promoting sustainable behavioral change, e.g., regarding stress management or daily physical activity, is central to inpatient rehabilitation.

Research indicates that successful transitions of insights and health-promoting strategies from rehabilitation into daily life and work can lead to sustained health improvements and is crucial to maintaining long-lasting effects of rehabilitation (Köpke, 2016). Incorporating these strategies into personal and professional life, however, is a challenge for many patients. Consequently, suitable aftercare following acute treatment is crucial to avoid long periods of illness and to secure long-term workability and employment (Köpke, 2016).

The German Pension Insurance Association offers multimodal (IRENA) and unimodal (Psy-RENA) face-to-face aftercare concepts that stabilize the results of inpatient treatment (Deutsche Rentenversicherung, 2023) and thus promote return to work. However, uptake is limited due to a lack of outpatient services, long travel distances and limited time due to professional or family obligations (Harfst et al., 2003; Sibold et al., 2011). Furthermore, established aftercare programs predominantly focus on the treatment of physical limitations (Huber et al., 2014; Schulz-Behrendt et al., 2017). Specific problem situations, such as high psychological stress, interpersonal conflicts, or the lack of support in the personal or work environment, which indicate a need for social, emotional, and personal support are often overlooked (Kobelt et al., 2011; Schulz-Behrendt et al., 2017). Important aspects, such as support in terms of goal orientation, coping with illness, and accepting transitional phases with lower income (Klaus et al., 2018) are not addressed sufficiently.

To meet the demands for aftercare services and provide support acknowledging the specific needs of patients, low-threshold internet-based aftercare interventions offer a promising addition to traditional face-to-face interventions. Given the rapid development and increased reliance on digital solutions during the COVID-19 pandemic, increased acceptance and uptake of digital health applications by patients can be expected (de Witte et al., 2020). Accordingly, internet-based aftercare interventions are increasingly incorporated into routine care.

Numerous studies and meta-analyses provide evidence for the efficacy and effectiveness of internet-based interventions in prevention, treatment, and aftercare for various mental disorders (Barak et al., 2008; Sander et al., 2016; Andersson et al., 2019) and the maintenance of treatment gains post-treatment (Hennemann et al., 2018; Kockler et al., 2021; Stavric et al., 2022), although outcomes related to return to work are rarely studied.

The aftercare program *GSA-Online plus* (Healthy and Stress-Free at Work), a psychotherapeutically oriented guided online writing intervention for occupationally stressed rehabilitants, was evaluated in a previous randomized-controlled-trial (RCT). Participants of the intervention group (IG) reported a significantly more positive subjective prognosis of gainful employment (*p* = .018) compared to the active control group (CG). Additionally, measures of depression (*p* = .018), anxiety (*p* = .025), and psychosocial stressors (*p* = .015) decreased in the IG from baseline to follow-up, while corresponding scores increased in the CG (Zwerenz et al., 2017).

In an implementation study in a routine care setting, *GSA-Online plus* was prescribed significantly less often than expected (Zwerenz et al., 2019). We argued that the low referral rate may be due to *GSA-Online plus* focusing specifically on the psychological comorbidity. Interventions providing more general support with a practical focus on social work may be of greater benefit to the diverse target population and better fit the multidisciplinary treatment approach that is a main characteristic of inpatient medical rehabilitation in Germany (Zwerenz et al., 2019).

Therefore, the present study aims to develop and evaluate a low-threshold internet- and mobile-based tool for individualized return to work planning which provides support for a larger group of patients. The self-guided progressive web-app *marena* (My Work-related Rehabilitation Aftercare) aims to support return to work after inpatient medical rehabilitation. The intervention provides rehabilitation patients with information on rehabilitation measures and common strategies known from inpatient rehabilitation. The intervention is designed to support the implementation of action plans and exercises in the patients' daily lives. While still in rehabilitation, users are encouraged to identify personal aftercare goals. Following discharge, *marena* provides weekly tasks to work on these goals and, if necessary, seek personal or professional support or revise aftercare goals. Additionally, based on information provided by the user, such as the end of medical rehabilitation, the first day at work after rehabilitation, or a reported event such as sickness during a graduated return to work measure, *marena* automatically and dynamically provides content and recommends actions relevant to the user's specific situation.

To meet patients' individual support needs, IG participants will be given access to the interventions in a stepped-care model. As the unguided *marena* intervention is expected to be meaningful and appropriate for all individuals fulfilling the inclusion criteria of the study, all participants assigned to the IG will have access to *marena*. Participants of the IG with a specific need for occupational-related treatment (assessed with the social medical risk index SIBAR and clinic staff recommendation) and a need for individual therapeutic guidance will have additional access to *GSA-Online plus*. Participants of the CG will only receive access to a survey feature within *marena.*

Participants with access to *GSA-Online plus* will be more likely to have an unfavorable occupational prognosis (e.g., increased risk of early retirement, longer periods of sick leave, and an increased burden of symptoms). However, through interacting with their online therapists in the guided *GSA-Online plus* intervention, participants may receive additional benefits compared to those who only have access to *marena*. Hence, participants may not differ significantly on the primary outcome of work status but may show differences on secondary outcomes. To control for baseline differences in the two experimental conditions, we will conduct exploratory evaluations concerning adherence, satisfaction, and efficacy.

Furthermore, we will examine personal and work related variables, which in previous research have shown an impact on return to work. These include work ability (Figueredo et al., 2020), physical well-being (Schmidt et al., 2011; Streibelt and Egner, 2013), social support (Etuknwa et al., 2019; Figueredo et al., 2020), depression and anxiety (Islam et al., 2014; Hesse et al., 2019; Figueredo et al., 2020; Techniker Krankenkasse, 2023), somatic symptoms (Brouwers et al., 2009; Momsen et al., 2014), and stress. Due to the central protective function of resilient coping against psychological crises, which is associated with functions important upon return to work, such as active problem solving, seeking social support, and higher perceived health competence, among others, we additionally include the assessment of resilient coping (Sinclair and Wallston, 2004). Furthermore, we will take a look at the subjective prognosis of gainful employment due to the predictive value on (early) retirement (Mittag and Raspe, 2003; Bürger and Deck, 2009), as well as the completion of graded return to work and benefits for participation in working life measures, both of which are frequently used in the German rehabilitation system and are supported by *marena*. Finally, we will ask participants to rate their satisfaction as well as the usability and helpfulness of the interventions.

It is hypothesized that participants in the IG (online self-help intervention *marena*) will report significantly higher levels of return to work (work status at the end of the intervention and 12 months after discharge from rehabilitation) compared to the optimized CG as primary outcome. Furthermore, we expect that participants in the IG will report significantly better work-related outcomes (successful completion of graded return to work, successful application for benefits for participation in working life, current work ability, subjective prognosis of gainful employment), quality of life, and resources (coping skills, social support), as well as significantly less distress (lower rates of social-medical risk index, somatic symptoms, depression, anxiety, and psychosocial stress as secondary outcomes). Additionally, the two IG subgroups (*marena* alone and *marena* in combination with *GSA-Online plus*) are further compared on an exploratory level with respect to the primary and secondary outcomes.

## 2 Methods

### 2.1 Study design

**Fig. 1:**
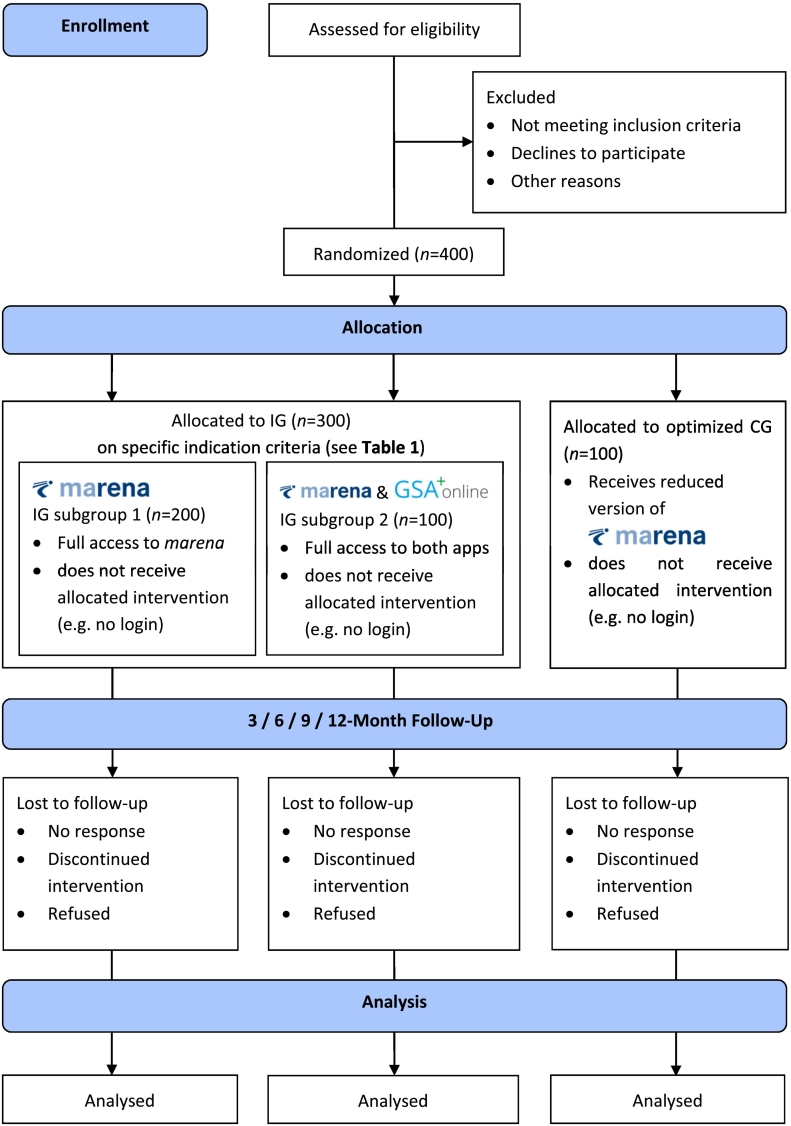
CONSORT-EHEALTH flow chart of the study design.

In the two-arm multicentered efficacy RCT, participants are allocated to either the IG or the CG at a 3:1 ratio by means of block randomization with varying block sizes stratified by rehabilitation center. The IG will get access to either the unguided *marena* (IG subgroup 1) or *marena* in combination with the guided *GSA-Online plus* (IG subgroup 2) based on their specific indication criteria (see Table 1). The CG will receive optimized treatment as usual with access to a survey feature within the *marena* application. A Consolidated Standards of Reporting Trials (CONSORT) flow diagram is shown in Fig. 1.

**Table 1:** Indication criteria.

Intervention	Indication criteria
IG subgroup 1	Patients … (a) in need of support in the process of transferring knowledge, skills and strategies acquired during inpatient rehabilitation into everyday life and work, or (b) in need of rehabilitation-related information regarding benefits, eligibility, legal issues and procedures, or (c) who need support and structured information on occupational reintegration (e.g., information about responsible authorities, forms, deadlines).
IG subgroup 2	Patients who additionally experience… (a) a specific need for occupational-related treatment (assessed with the social medical risk index SIBAR) or (b) subjectively high psychological stress (anxiety, feelings of being overwhelmed) related to returning to work, or (c) social stress or interpersonal conflicts at work, or (d) a high subjective need for individual therapeutic guidance in (c.1) dealing with stressful situations at work and developing personal coping resources or in (c.2) coping with recurrent unfavorable interpersonal interactions at the workplace.

### 2.2 Recruitment and eligibility criteria

A total of *N* = 400 rehabilitants are planned to be included in the study. Participants with various indications will be recruited during inpatient psychosomatic, orthopedic, or cardiological treatment in four rehabilitation centers in Germany. Rehabilitation entails an intensive examination, information about the individual's disease or disability, and the definition of rehabilitation goals to be achieved through various on-site measures. Patients are taught coping strategies to help them cope with the illness or disability in everyday life after rehabilitation. Since the majority of patients in rehabilitation are currently unable to work or are at risk of reduced earning capacity, this also entails dealing with occupational problems. Medical rehabilitation typically lasts 3 weeks. Psychosomatic rehabilitation typically last five or six weeks.

All rehabilitants are screened for eligibility based on clinical routine data through the clinic staff of the rehabilitation centers. The inclusion criteria are: (1) planned return to work (full- or part-time); (2) age 18 to 65 years; (3) (undisturbed) access to a computer/mobile device and the internet; and (4) German Pension (federal or regional) or statutory health insurance as rehabilitation institution. The exclusion criteria are (1) current unemployment or reduced earning capacity pension with no immediate prospect of employment and (2) negative prognosis for employment (ability to work on the general labor market <3 h as assessed in rehabilitation). No specific diagnoses will be excluded from participation.

Eligible rehabilitants will be personally informed in a group presentation by the clinic staff or members of the study team. Furthermore, they will be provided written study information and a study flyer with a link to the study homepage. Upon receipt of the signed informed consent, clinical staff will assess whether IG subgroup 1 or IG subgroup 2 is indicated and provide the study team with the recommendation for an intervention subgroup and data needed for enrollment. Table 1 provides a listing of the indication criteria for each subgroup. Indication criteria were defined based on the *GSA-Online plus* implementation study and the clinics' assessment of the expected support needed by patients. We anticipate that one-third of eligible participants will get a referral for *GSA-Online plus*. The study team will randomly assign participants to the IG or CG and assign IG participants to the recommended subgroup. After randomization, participants will receive an e-mail with initial data to log into the allocated interventions.

### 2.3 Randomization

Participants will be randomized to either IG or CG in a 3:1 ratio, stratified by rehabilitation center. Randomization lists with permuted blocks of variable lengths will be created. Upon enrollment of participants by the clinic staff, the study team will access the respective randomization list provided by an independent researcher not otherwise involved in the study. If a participant is allocated to the CG, this is entered within *marena*. If a participant is allocated to the IG, the subgroup recommended by the clinical staff is entered within *marena.* Participants will be informed about their group assignment in the registration e-mail. Due to the nature of the interventions, blinding of the group assignment is not possible.

### 2.4 Interventions

Initial login data for the interventions is received via e-mail while still in inpatient rehabilitation. Participants will be granted access after setting an individual username and password.

#### 2.4.1 marena

Participants of the IG have access to the unguided self-help intervention *marena* for a period of 6 months following discharge from rehabilitation. The content of *marena* is based on comprehensive research of respective literature. This includes research on well-established strategies in inpatient treatment, as well as information on financial and practical measures relevant to the rehabilitation process. These strategies range from audio exercises covering relaxation techniques (Deutsche Rentenversicherung Bund), emotional awareness (Martins et al., 2010; Kraiss et al., 2020), and mindful eating (Robinson et al., 2013; Warren et al., 2017) to information on the benefits of physical activity (Pedersen and Saltin, 2015), work breaks and leisure-time (Sonnentag et al., 2010; Hunter and Wu, 2016; Bennett et al., 2018; Wiese et al., 2018), and goal-setting (Schumacher; Maclean and Pound, 2000). Information on financial and practical measures relates, for example, to graded return to work (Deutsche Rentenversicherung Bund, 2023a), benefits for participation in working life (Deutsche Rentenversicherung Bund, 2018), reintegration management (Deutsche Rentenversicherung Bund, 2023c), and financial aid during sickness and rehabilitation (Deutsche Rentenversicherung Bund, 2023b; §§ 44 ff. SGB V).

Furthermore, structured interviews (*N* = 11) were conducted with experts from research and practice to determine the specific focus of the intervention. Case groups based on work-related rehabilitation goals were defined and frequent, prototypical pathways and associated obstacles were identified. Features and contents were adapted through constant feedback loops with the experts interviewed beforehand.

Consequently, the intervention consists of four main areas: (1) a task organizer where participants can create their own and receive tasks according to three different aftercare plans, (2) a strategy area with summaries and audio exercises for a collection of strategies known from rehabilitation (e.g., physical activity and relaxation techniques), (3) frequently asked questions with specific information on financial and practical measures (such as sickness benefit and graded return to work) and (4) a contact directory.

Ultimately, a prototype of *marena* was piloted with *N* = 7 rehabilitation patients. The qualitative Think Aloud method was used in semi-structured interviews with patients accessing the *marena* intervention on their private mobile devices. Participants performed different tasks based on the key features in each section of the intervention and were asked to verbalize their thoughts while performing tasks and activities. Recommendations for improvement were then made based on participants' comments about the content, appearance, or functionality of *marena*. The intervention was then updated accordingly.

A (1) return to work aftercare plan is preselected and requests participants to specify aftercare goals while still in inpatient rehabilitation. The intervention encourages participants to discuss their aftercare goals with health professionals in the rehabilitation centers. Following discharge from inpatient rehabilitation, *marena* provides weekly tasks to work on these goals and refers to other related areas of the intervention (e.g., topics such as motivation, mindfulness, or physical activity in the strategy area). Participants can additionally specify if they (2) undertake graded return to work or apply for (3) benefits for participation in working life. The graded return to work aftercare plan contains tasks with information on the procedure as well as reminders on important appointments and forms. The benefits for participation in working life aftercare plan contains tasks that guide participants through the application process with references to necessary information and application forms. Facing typical obstacles or setbacks during occupational reintegration, participants can report changes in return to work dates, sickness within graded return to work, and different responses to a benefit for participation in working life application. The intervention provides corresponding tasks with recommended actions and contacts. Activity, due date, and content of tasks are dependent on patients' profile data. Each plan provides relevant contacts.

Within *marena* participants are offered a voluntary weekly survey. This survey includes three items on health status (EQ-VAS), workload (SIBAR subscale occupational stress), and ability to work (WAS). If participants regularly complete these surveys, they can view a corresponding graph for each item in their profile over the course of program participation.

**Fig. 2:**
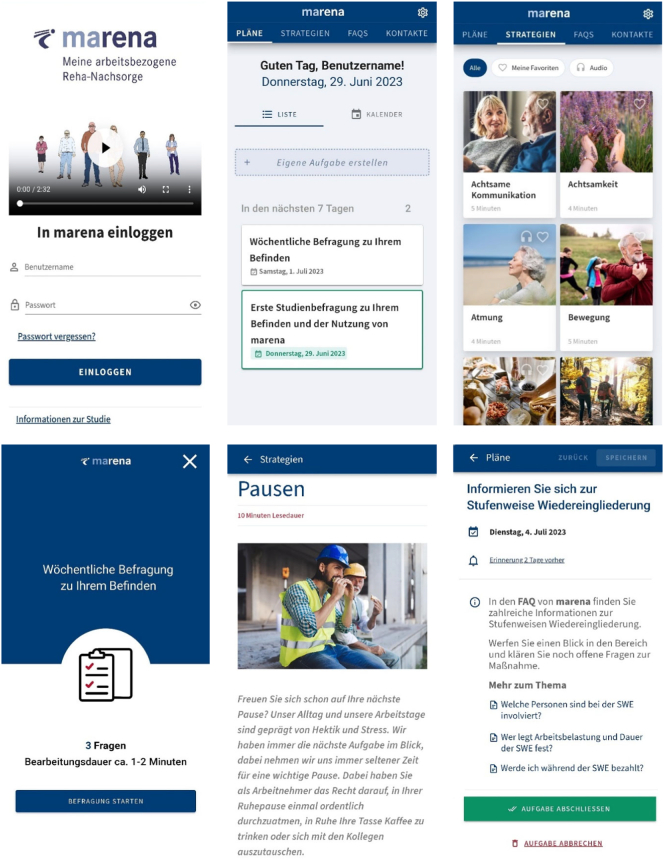
Excerpts from the *marena* intervention.

Participants receive reminders on new due tasks, study surveys, and after 14 days of inactivity. Depending on the operating system, reminders will be sent via e-mail and/or push notification. For excerpts from the *marena* intervention, see Fig. 2.

#### 2.4.2 GSA-Online plus

*GSA-Online plus* is an internet-based psychotherapeutically oriented aftercare intervention (Beutel et al., 2018) which aims to support occupationally stressed rehabilitants during return to work. The efficacy and feasibility of the therapeutic writing intervention has already been demonstrated in an RCT (Zwerenz et al., 2017) and an implementation study (Zwerenz et al., 2019).

While still in inpatient rehabilitation, participants can access explanatory and motivational videos to familiarize themselves with the program and select a specific day of the week to receive writing prompts. Following discharge, participants receive weekly writing prompts over a period of 12 weeks. The participants respond with a diary entry and are instructed to articulate and identify interpersonal and intrapsychic problems at the workplace. The online therapists reply to the diary entries by providing encouragement and support to deal with individual job-related problems, focusing on stabilizing work capacity.

The first writing prompt is standardized. The following prompts and feedback are personalized, depending on past diary entries. Diary entries are accessible only to the individual participant and the study team. Communication takes place only within the intervention. Participants receive e-mail reminders on new, due, and overdue writing prompts as well as when feedback from the online therapist is available.

### 2.5 Control condition

Participants in the CG will have limited access to *marena* with questionnaires and voluntary weekly surveys. We chose to use an optimized control condition in anticipation of higher participation during recruitment at the rehabilitation centers and higher response rates to follow-up assessments. Participants in both intervention arms will have unrestricted access to usual treatment options.

### 2.6 Outcome measures

Outcomes are assessed at the end of rehabilitation (T0) as well as three (T1), six (T2), nine (T3), and 12 months after discharge (T4). Table 2 provides an overview of the data collected at the five measuring points. We carefully selected compact instruments to reduce the burden on the participants.

**Table 2:** Schedule of assessments.

Construct	Questionnaire	T0	T1	T2	T3	T4
Primary Outcome						
Work status	employment status at 6 and 12 months of follow-up	x	x	x	x	x

Secondary Outcomes						
Successful completion of a graded return to work	Self-constructed items	x	x	x	x	x
Successful application for benefits for participation in working life	Self-constructed items	x	x	x	x	x
Current work ability	WAS^a^	x	x	x	x	x
Social-medical risk index	SIBAR^a^	x	x	x	x	x
Health related quality of life	EQ-5D^a^	x	x	x	x	x
Social Support	OSSS-3	x	x	x	x	x
Coping	BRCS	x	x	x	x	x
Anxiety	GAD-7	x	x	x	x	x
Depression	PHQ-9	x	x	x	x	x
Psychosocial Stress	PHQ-Stress	x	x	x	x	x
Somatic symptoms	SSS-8	x	x	x	x	x
Subjective prognosis of gainful employment	SPE	x	x	x	x	x
Satisfaction with *marena*	ZUF-8 *marena*^b^, ^d^			x		
Usability of *marena*	UEQ *marena*^b^, ^d^			x		
Helpfulness of *marena*	Self-constructed items^b^, ^d^			x		
Satisfaction with *GSA-Online plus*	ZUF-8 *GSA-Online plus*^c^, ^d^		x			
Usability of *GSA-Online plus*	UEQ *GSA-Online plus*^c^, ^d^		x			
Helpfulness of *GSA-Online plus*	Self-constructed items^c^, ^d^		x			

Other measures						
Demographics	Socio-Demographic Data	x				
Treatment data	Clinical Routine Data	x				
Occupational data	Clinical Routine Data	x				
Recommendations for rehabilitation aftercare	Clinical Routine Data	x				
General Feedback	Self-constructed items^b^		x	x		x
Adherence *marena*	Number of sessions in the intervention^b^, ^d^ Number of completed tasks^b^, ^d^ Number of completed weekly survey^b^, ^d^ Number of media playback^b^, ^d^ Number of visits to the intervention areas and individual pages^b^, ^d^	n. a.	n. a.	n. a.	n. a.	n. a.
Adherence *GSA-Online plus*	Number of completed writing-impulses^c^, ^d^	n. a.	n. a.	n. a.	n. a.	n. a.

a Work ability status, health condition and occupational stress are additionally provided in a weekly survey, so they are regularly answered during the intervention phase.

b Only participants of IG subgroup 1.

c Only participants of IG subgroup 2.

d Objective data collected within the programs.

#### 2.6.1 Primary outcome

The primary outcome will be work status at six (T2) and 12 (T4) months follow-up assessment. Work status at 6 months will be considered as primary endpoint and is defined as positive if the participant is working (no unemployment, no reduced earning capacity pension) and reports a maximum of six weeks of sick leave not related to graded return to work. Work status at 12 months is defined as positive if the participant is working and reports maximum 12 weeks of sick leave not related to graded return to work. These cut-offs were chosen in line with previous analyses of return to work in German rehabilitation (Mau et al., 2002; Bethge et al., 2011). We do not include sick leave during a graded return to work measure, as participants are formally on sick leave, receiving financial support from their healthcare provider, but are back at their workplace.

#### 2.6.2 Secondary outcomes

Successful completion of a graded return to work is assessed with self-constructed items about the planning, execution, completion, discontinuation, and early termination of the measure.

Successful application for benefits for participation in working life is assessed with self-constructed items about the planning, execution, and completion of an application, as well as the (partial) approval or rejection of benefits.

Current work ability is assessed with the Work Ability Score (WAS), the first single-item question in the well-established Work Ability Index (WAI; Hasselhorn and Freude, 2007). Participants rate their current work ability compared with their lifetime best. The scale ranges from 0 to 10.

Social-medical risk index is assessed with the short and validated ‘Screening Instrument for the Assessment of Need for Occupational Related Treatment in Medical Rehabilitation’ (SIBAR; Bürger and Deck, 2009) which comprises three subscales: (1) the social-medical index (risk of early retirement); (2) occupational stress, and (3) the subjective need for occupation-related treatment. We will assess scale 1 and scale 2. The social-medical index ranges from 0 to 19. A need for occupational treatment is indicated if the score is 8 or higher. The single global rating on occupational stress of subscale 2 is: ‘Overall, my professional situation is… very stressful… to … very fulfilling’ measured on a five-point Likert scale from 0 to 4. The retest-reliability of the different subscales ranges from rep = 0.37 to rep = 0.96 in a previous study by Bürger and Deck (2009).

Subjective prognosis of gainful employment is assessed by a 3-item scale measuring the subjective prognosis of future work capacity and employment status (SPE scale; Mittag and Raspe, 2003).

Health related quality of life is assessed through the European Quality of Life Questionnaire (5 Dimensions; EQ-5D; Ludwig et al., 2018). The instrument includes a vertical EQ Visual Analogue Scale (EQ-VAS), which provides a single global rating of self-perceived health. The scale is measured on a Likert scale from 0 to 10 (0 = ‘worst imaginable health’ to 100 = ‘best imaginable health’).

Somatic symptoms are assessed with the symptom scale-8 (SSS-8; Gierk et al., 2014). The 8 item scale assess different symptoms on a Likert-scale ranging from 0 = ‘not at all‘ to 4 = ‘very much’. An example item is ‘During the past 7 days, how much have you been bothered by back pain?’.

Coping is assessed by the Brief Resilient Coping Scale (BRCS). An example out of the four items is: ‘Regardless of what happens to me, I believe I can control my reaction to it’. The Likert-scale ranges from 1 = ‘does not describe me at all’ to 5 = ‘describes me very well’.

Psychosocial Stress is assessed with the subscale ‘PHQ-stress’ from the Patient Health Questionnaire (PHQ-D; Löwe et al., 2002). An example item is: ‘How much did you feel impaired by stress at work or school in the last 4 weeks?’. The 10 items include health, work/financial, social, and traumatic stress and the ratings comprise 0 = ‘not at all bothered’, 1 = ‘bothered a little’, and 2 = ‘bothered a lot’.

Depression is assessed with the Depression Module (PHQ-9) of the PHQ-D (Spitzer et al., 1999). An example item of this scale is: ‘Over the last two weeks, how often have you been bothered by feeling tired or having little energy?’. The response format is a 4-point Likert-scale ranging from 0 = ‘not at all’ to 3 = ‘nearly every day’.

Anxiety is assessed with the Generalized Anxiety Disorder Scale-7 (GAD-7; Spitzer et al., 2006). An example item is: ‘Over the last two weeks, how often have you been bothered by not being able to stop or control worrying?’. The response format ranges from 0 = ‘not at all’ to 3 = ‘nearly every day’ and corresponds to a 4-point Likert-scale.

Social Support is measured by the Oslo Social Support Scale (OSSS-3; Dalgard and Brevik, 1996). A total of three items assess the level of perceived social support. The response format varies from item to item. Both 4-point and 5-point Likert-scales are used. Higher values indicate greater social support. An example item is: ‘How much interest and concern do people show in what you do?’.

Satisfaction with treatment is assessed with a modified version (wording adapted for the online-interventions) of the Client Satisfaction Questionnaire (CSQ-8; Attkisson and Zwick, 1982). To adapt the questionnaire for our sample, the German version called ZUF-8 by Schmidt et al. (1989) will be used. The Likert-scale ranges from 1 to 4, with higher values indicating higher satisfaction. An example item is: ‘Overall, how satisfied are you with the treatment you have received?’.

User Experience is assessed using the 8-item User Experience Questionnaire (UEQ; Schrepp et al., 2017). The Likert-scale ranges from 0 to 6 with two poles each, for example, 0 = ‘confusing’ and 6 = ‘clear’.

Helpfulness is assessed with quantitative and qualitative self-constructed items. Participants will rate overall helpfulness on a 5-point Likert-scale ranging from 1 = ‘very much’ to 4 = ‘not at all’. Furthermore, participants are asked to indicate which elements of the interventions were particularly helpful/not helpful.

#### 2.6.3 Other assessments

Demographic and socioeconomic characteristics from clinical routine data include age, sex, marital status, level of education, professional qualifications and apprenticeships or studies.

Occupational data include weekly working hours, receipt or application for a pension, unemployment at the time of admission, and incapacity to work in the last 12 months before admission to rehabilitation.

Inpatient treatment data includes admission and discharge dates, discharge type, and ICD 10 diagnoses.

Recommendations for medical procedures and rehabilitation aftercare by the rehabilitation clinic are documented.

General feedback is assessed through a self-constructed item (‘What else would you like to tell us?’).

Adherence is assessed in different areas throughout *marena*. To analyze the use of the task organizer the number of completed tasks will be examined in relation to the number of tasks that became active in the participant's aftercare plan. Adherence is operationalized by the percentage of participants who completed at least the lower quartile of the provided tasks (in relation to the sample). Additionally, the number of sessions, completed weekly surveys, media playback, visits to the intervention areas, and individual pages will be analyzed. To operationalize adherence to *GSA-Online plus*, the number of completed writing-impulses will be assessed. Adherence will be operationalized by the percentage of participants who wrote at least the lower quartile of diary entries written in the sample.

### 2.7 Sample size

The sample size was determined a priori using SAS 9.4 software. A Power analysis based on a power of 0.80, two-sided testing, an alpha-rate of 5 %, and a randomization ratio of 3:1 was conducted. According to Bethge (2017), Odds Ratios of 3.49 (referring to Streibelt and Bethge, 2014) and 2.41 (in Bethge et al., 2011; overall in Streibelt and Bethge, 2014) were observed for work-related occupational rehabilitation programs versus medical rehabilitation with regard to work status 6 months after discharge from rehabilitation. We plan more conservative with an Odds Ratio of 2.0. According to Bethge (2011), a work status rate of 51 % in the CG could be assumed. These assumptions resulted in a total sample size of *N* = 360 required for 80 % power and a binary logistic regression model to analyze the primary endpoint. When considering a correlation of ρ = 0.4 with indicators of specific occupational problems (at least 3-months of incapacity to work before rehabilitation, unemployment at the time of application, and the socio-medical assessment of limited performance in the last occupational activity; see also Bethge, 2017), the sample size can be reduced by ρ2 = 16 % to *N* = 320, due to the reduction of total variance through the explained variance of the covariates. Based on an estimated dropout rate of 20 % at the follow-up assessments T1 (at the end of the *GSA-Online plus* intervention) and T2 (at the end of the *marena* intervention) we plan to randomize *N* = 400 patients.

### 2.8 Data collection methods

The total planned duration of the RCT is 24 months, including 12 months of recruitment and 12 months of follow-up after inpatient rehabilitation. Data collection for the main outcome variables will be conducted through online surveys within *marena*. Routine data from rehabilitation treatment will be used for screening and, if participants are eligible, collection of demographic and enrollment information. Study assessments will take place at the end of rehabilitation (T0), as well as at three (T1), six (T2), nine (T3), and 12 months after discharge (T4). Additionally, voluntary weekly surveys can be submitted.

### 2.9 Statistical analysis for primary and secondary outcomes

All data analyses will be performed after completion of data collection and on intention-to-treat basis. The significance level for all analyses will be defined as *p* = .05. If appropriate, results will be displayed by adjusted estimates from the analysis model, and their respective 95 % confidence intervals. Descriptive statistics will be used to describe demographics and baseline characteristics. To examine differences between IG and CG in the dichotomous primary outcome work status at 6 months (T2, primary endpoint) and 12 months (T4, secondary endpoint), a binary logistic regression model with fixed and random effects will be performed. In the logistic regression model the following parameters will be considered as fixed effects: treatment group (IG/CG), study site, operating system (android/iOS), at least 3-month incapacity for work before rehabilitation (yes/no), and unemployment at the time of admission to rehabilitation (yes/no). The social-medical risk index (SIBAR) as a continuous parameter will serve as a random effect. In addition, per protocol analyses will be performed to examine the impact of dropouts on study results. Dichotomous secondary outcomes will be analyzed accordingly. Continuous outcome measures will be analyzed with a covariance model for repeated measures. Compound symmetry will be assumed. Additionally, per protocol analyses (based on data of those participants who completed at least the lower quartile of tasks provided in *marena* in relation to the sample and participants who wrote at least the lower quartile of diary entries written in the *GSA-Online plus* sample) will be performed to examine the impact of dropouts on study results. Furthermore, other possible influencing factors (e.g., IG subgroup 1 and IG subgroup 2, received aftercare vs. not received aftercare) will be examined in an exploratory way. The results will also be displayed by rehabilitation center to explore whether obvious differences in effect sizes by rehabilitation center are apparent.

## 3 Discussion

This RCT examines the efficacy of the newly developed internet- and mobile-based aftercare intervention *marena* concerning work status as the primary outcome, the success on work-related measures (graded return to work and benefits for participation in working life), current work ability, as well as psychosocial well-being as secondary outcomes. We will also examine aspects of acceptability, such as intervention satisfaction and adherence, to improve further development of the intervention. Rehabilitants with access to *marena* are compared to an optimized CG with limited access to the intervention. If the rehabilitation clinic staff identifies a need for more intensive and specific support in addition to *marena*, participants of the IG are given access to the web-based guided intervention, *GSA-Online plus.*

The *marena* intervention is designed as a low threshold application intended to support return to work following inpatient medical rehabilitation. To ensure that participants receive relevant information depending on how and when they return to their workplace, and which supporting measures are available, adaptable content and various components customized to the participants' input are provided. Content tailoring allows participants to make choices and report events in real time, which in turn, triggers different content based on preference or need. Results will contribute to the limited evidence on internet-based interventions focused on return to work in occupational health (Johnsen et al., 2021).

### 3.1 Strengths

First, the intensive development phase for *marena* with expert consultation and feedback loops, as well as a pilot phase with patients to ensure the content is tailored to the target group and the intervention is user-friendly. This could also have a positive impact on adherence and on subsequent implementation in the healthcare system.

Second, the intervention aims to facilitate the transfer of customary strategies and aftercare goals into daily life, with content closely linked to inpatient and occupational rehabilitation. To accomplish this, specific aftercare plans are assigned, that contain sequences of tasks to be performed by the rehabilitants. If applicable to the participants, these plans include tasks to fulfill their legal obligations (e.g., sending a form to the pension insurance agency within the framework of a graded return to work). The aftercare plans are developed in a declarative way, allowing the activation and due dates of all tasks to be automatically and dynamically adapted to the development of the participants' status, thus providing unsupervised personalization of the participants' plans. By providing structure and guidance through additional information and encouragement, *marena* supports the implementation of action plans from inpatient rehabilitation into daily life, motivates participants to pursue their aftercare goals, and aims to achieve a sustainable anchoring of the successes of rehabilitation in subsequent work and everyday life.

Third, the stepped-care model with different levels of guidance ensures that the individual need for support is met. Internet-based aftercare provides the opportunity to reach patients independently of time and place. While guided approaches have shown to be most effective, they are also tied to greater intervention cost (Baumeister et al., 2014). Thus, investigating the efficacy and acceptability of an unguided aftercare intervention is particularly valuable.

Finally, we will be able to enroll a variety of patients in the target population of this study by recruiting across indications, in four different states, and in clinics with different health care providers (two German Pension insurance clinics, two private clinic operators). Participants will not be compensated for their participation. This real-world setting, reflecting the conditions of health care services, will add considerable validity to future findings and will provide very valuable information on the generalizability of study results as well as the long-term implementation and dissemination of both interventions.

### 3.2 Limitations

First, we emphasize that there is no randomization between the two IG subgroups, as recommendations are made by the clinic staff. Therefore, selection bias may limit the generalizability of the results. To reduce bias, standardized training and information materials are provided to ensure that the selection of participants is as objective as possible.

Second, due to the nature of the intervention, study participants will not be blinded. Participants in the CG, who will not have access to the full version of *marena*, will be aware when reading the consent form and attending the group presentation, fellow patients will have access to a more favorable version of the intervention. To mitigate this potential disincentive, we decided to use an optimized CG.

Third, it should also be noted that content in *marena* supports participants during a graded return to work, where participants remain on formal sick leave, and thus provides content that actively leads to inability to work. This may have a negative impact on the primary outcome work status. To reduce the risk of confounding of the criterion, we will not include work incapacity due to graded return to work in the outcome. Furthermore, including the duration of work incapacity in the screening instrument as well as the primary outcome may also confound the results.

Furthermore, a high dropout rate is to be expected, as internet-based interventions are typically subject to low adherence, especially in the CG. Therefore, multiple features of *marena* are designed to encourage compliance and questionnaire response, such as task reminders and questionnaires that are set to be active for a period of time and become mandatory close to the measurement points.

Last, the different functionality of *marena* on the Android (push notifications and e-mail) and iOS (e-mail only) operating systems may contribute to differences in user compliance. To control for this, the operating system used is included in the analysis as a control variable.

### 3.3 Conclusion

Patients returning to work following a medical rehabilitation face a diversity of challenges. Providing tangible and practicable advice regarding the pursuit of aftercare goals, aftercare programs/measures and benefits. If it proves to be effective, *marena* offers a highly adaptive, low-threshold approach to improve return to work in rehabilitation patients.

## CRediT authorship contribution statement

AK, RZ, AM, CR and MB designed the study. AK, RZ and ID developed the *marena* intervention. AS, GK, ES, VS, GL, HZ, TS and ML elaborated and defined the requirements of the *marena* intervention and provided feedback for on the developed intervention components. ID is responsible for the software development. GK, ES, VS, GL, HZ, TS and ML contribute to data collection and provision at the collaborating rehabilitation centers. AK, HS and RK wrote the first draft of the manuscript. RZ, MB, AM, AT, ID, CR and AS revised the manuscript critically. All authors contributed feedback, read, and approved the final manuscript. With regard to the requirement for authorship, the working group follows the guidelines for the assurance of good scientific practice of the German Research Foundation (2022). This article was written as part of the doctoral thesis of AK.

## Trial registration

The trial is registered at the German Clinical Studies Trial Register (registration number DRKS00029382). Participants are recruited from July 2023 until July 2024.

## Research ethics approval

Ethical approval for the study was obtained from the Ethics Committee of the Federal State of Rhineland-Palatinate (registration number 2021-16119), Baden-Württemberg (reference number B-F-2023-038), and North Rhine-Westphalia (registration number 2023-307-b-S). The Ethics Committee of the Federal State of Bavaria was informed about the study. Participants will receive written information about the study conditions, data security, voluntary nature of participation, and the right to withdraw consent and have data deleted.

## Funding

The study is funded by the German Statutory Pension Insurance (DRV-Bund; grant number AZ 8011-106-31/31.134). The funding source had or has no involvement in the study design, management, analysis and interpretation of data, or the decision to submit results.

## Declaration of competing interest

The authors declare the following financial interests/personal relationships which may be considered as potential competing interests: Adina Kreis reports financial support was provided by German Pension Insurance. Ruediger Zwerenz reports financial support was provided by German Pension Insurance. Manfred Beutel reports financial support was provided by German Pension Insurance. Anna Gomes reports financial support was provided by German Pension Insurance. The authors are the evaluators and developers of the software. The cooperating clinics are compensated for recruitment.
